# What can shopping transactional data reveal about relative prevalence of menstrual pain and period poverty in England?

**DOI:** 10.1371/journal.pdig.0001308

**Published:** 2026-05-28

**Authors:** Victoria Sivill, Vanja Ljevar, James Goulding, Anya Skatova

**Affiliations:** 1 The Alan Turing Institute, London, United Kingdom; 2 Intelligent Systems Laboratory, Department of Computer Science, University of Bristol, Bristol, United Kingdom; 3 N/LAB, Nottingham University Business School, University of Nottingham, Nottingham, United Kingdom; 4 Digital Footprints Lab, Population Health Sciences, Bristol Medical School, University of Bristol, Bristol, United Kingdom; 5 The MRC Integrative Epidemiology Unit at the University of Bristol, Population Health Sciences, Bristol Medical School, University of Bristol, Bristol, United Kingdom; The University of Sheffield, UNITED KINGDOM OF GREAT BRITAIN AND NORTHERN IRELAND

## Abstract

91% of those who menstruate reported to experiencing associated pain. Despite the ubiquity of this phenomenon, the prevalence, extent and sociodemographic variation of menstrual pain remains understudied at national levels. In this paper, we assess the extent and variation of menstrual pain at a national level. We develop a novel proxy measure for menstrual pain, utilising behavioural data extracted from supermarket shopping logs. Baskets where pain and menstrual items co-occur are investigated, and normalized against baskets in which pain or menstrual items occur in isolation. Propensity of menstrual pain purchases are aggregated temporally and geographically across England, prior to linkage with sociodemographic indicators regionally. Findings indicate high prevalence of menstrual pain across England, with 26.7% of customers in our dataset buying pain relief together with menstrual products. People who menstruate are observed to be four times more likely to purchase pain relief with menstrual items than without. Average regional income provides the strongest predictor of menstrual pain co-purchases, with lower income regions exhibiting a 32% lower menstrual-pain purchase than higher income regions. The robust presence of a consistent 28-day cycle in menstrual-pain purchases provides empirical evidence for the use of behavioural proxies for menstrual pain alongside traditional measures. Significant regional differences observed in the prevalence of menstrual-pain transactions across England brings into light existing disparities. Future research into improved understanding of sociodemographic factors associated with menstrual pain could inform strategies to predict and prevent menstrual pain and its adverse impacts.

## Author summary

Menstrual pain is extremely common, affecting more than nine in ten people who menstruate, yet it is rarely measured at a national level. This study explores how widespread menstrual pain is across England, and how it varies between different regions and groups. Instead of using surveys, the researchers created a new way to estimate menstrual pain by analysing supermarket shopping data. They looked for shopping baskets that contained both menstrual products and pain-relief medicines, comparing them with baskets that contained only one or the other. The results show that menstrual pain is highly prevalent: 26.7% of customers bought pain relief together with menstrual products. People who menstruate were four times more likely to buy pain relief when purchasing menstrual items than at other times. Income levels also played a role. Regions with lower average income showed 32% fewer menstrual-pain related purchases than higher-income regions, suggesting differences in access, need, or purchasing behaviour. The data showed a consistent 28-day cycle in these purchases, supporting the use of behavioural data as a reliable indicator of menstrual pain. Overall, the findings highlight meaningful regional differences and point to the need for further research to better understand and address menstrual pain and its impacts.

## Introduction

Experience of menstrual pain is an extremely common occurrence [1]. Contemporary evidence indicates that an 70–90% of women suffer from menstrual pain [2,3,4], with recent UK-based surveys reporting prevalence towards the upper end of this range [5]. Even though there can be several types of menstrual pain, the condition is commonly referred to as *dysmenorrhea*, a cramp- like pain in the lower abdomen occurring before and during menstrual period [1]. A range of symptoms exist that are subsequent to menstrual pain, including nausea, headache, vomiting, diarrhoea, fatigue and appetite loss [2,6]. This experience brings many negative consequences both socially and economically, as well as to the individuals themselves: limited daily activity, school or work absenteeism, negative impact on health-related quality of life, along with a significant health care expenditure [7,8].

Despite the ubiquity of menstrual pain, it has seen only very limited attention in the scientific literature, especially in terms of population level estimates. Women often take (and encouraged to take by society and medical professionals) a stoic approach when experiencing menstrual pain. Worryingly, even though menstrual pain can be debilitating, evidence indicates that not all women seek medical help for such symptoms: various studies have documented that substantial proportions of women either choose self-management or delay formal healthcare, often due to normative beliefs, concerns about treatment, or barriers in accessing care [9,10,11]. Reasons for this lie in the fact that menstrual pain is a complex sociocultural phenomenon, shaped by unhelpful set of perceptions [12]. Firstly, menstrual pain is largely perceived as a ‘part and parcel’ of female life [9]. Secondly, menstrual pain is often not seen as an a legitimate health issue, which lead to both women not having legitimate reasons to adopt appropriate illness behaviours [13,14]. To conclude, menstrual pain has a complicated perception of being ‘normal’ for women, but also’abnormal’, shameful and embarrassing, both of which bring many negative consequences and emotional stress for women and girls [12,14,15].

One of the main reasons why these perceptions still strive is because previous studies mostly had small sample sizes and consequently, detecting patterns of menstrual pain and period-poverty on national scales has been extremely challenging, slowing down decision-making in terms of policy decision and pharmacy services. This study considers these challenges through use of using geo-spatial models, based on a unique measure of menstrual pain extracted from consumption patterns identified in mass transactional datasets, in order to respond to two main questions:

- *What is the prevalence of menstrual pain, as inferred from over-the-counter self-medication and how does it vary geographically across England?* and
- *Are there differences in terms of sociodemographic profiles of those purchasing pain relief during their period and those who do not?*

To address these questions, we explore the feasibility of using transactional data to capture information about the prevalence and sociodemographic correlates of menstrual pain. We create transactional proxy measures for menstruation and menstrual pain to probabilistically evaluate the extent to which both conditions manifest across England, over a period of 10 years.

This research also investigates the relationship between menstrual pain and deprivation, utilising area-level measures of deprivation and socioeconomic position as proxies. By examining the MSOA (Middle Layer Super Output Area)-level transactions and publicly available social deprivation factors, the study aims to identify and classify regions in England as either high or low in terms of menstrual pain. We further used a feature importance mechanism to determine the most associated deprivation feature within our analysis.

## Methods

### Ethics statement

Ethics approval for the study was via the University of Nottingham ethics panel (Faculty of Social Sciences, Nottingham University Business School, review reference: 201829095). The data was obtained anonymously hence obtaining participants’ consent was not feasible.

### Sample

This study leverages mass shopping data records sourced from one of the UK’s leading health and beauty retailers, alongside a publicly available dataset that measures relative deprivation of neighbourhoods the length and breadth of the UK. The shopping dataset is made up of 211,332,752 unique loyalty-card transaction logs, reflecting purchases made by a total of 3,413,949 individuals over a ten-year period between 30th April 2006 and 16th April 2015. Within the transactional dataset used in this study, we implement a strict definition of a *menstrual product* as any item categorised by the retailer as “tampon” or “sanitary pad”. Applying this to the retailer’s product list returns a set of 548 Menstrual Products which feature in the transactional logs. We provide a similarly strict definition of a *pain product* as any item categorised by the retailer as either “Pain Relief Oral” or “Topical Pain Relief”, returning a set of 1018 items across the data. The 20 most frequently purchased menstrual and pain items are detailed in Tables 1 and 2 respectively.

**Table 1:** Top 20 menstrual products which cover 35% of the total menstrual products sold by the retailer between 30^th^ April 2006 and 16^th^ April 2015 (ST = sanitary towels, T = tampons).

MP Description	Percentage of Sales	Category	Unit Price	Overall Price
Always Ultra normal plus with wings 14s	3.62	ST	0.13	1.77
Always Ultra night wing towels 10s	2.99	ST	0.18	1.84
Always Ultra long plus with wings 12s	2.96	ST	0.15	1.84
Tampax Compak tampons regular 20s	2.05	T	0.13	2.67
Bodyform Ultra normal wing towels 14s	2.05	ST	0.10	1.40
non app tampons regular 16s	1.78	T	0.07	1.13
Always Ultra normal 16s	1.75	ST	0.11	1.79
Ultra towels normal 16s	1.60	ST	0.07	1.07
Ultra towels normal wing 14s	1.60	ST	0.07	1.03
Tampax Compak tampons super 20s	1.57	T	0.13	2.60
non app tampons super 16s	1.48	T	0.07	1.11
Ultra towels night wing 10s	1.44	ST	0.11	1.12
Lil-Lets tampons super plus extra 14s	1.38	T	0.19	2.30
Ultra towels super wing 12s	1.32	ST	0.09	1.04
applicator tampons regular 12s	1.30	T	0.09	1.02
Bodyform Ultra goodnight wing towels 10s	1.28	ST	0.15	1.46
Tampax Compak active tampons regular 20s	1.20	T	0.33	2.68
Ultra towels super 14s	1.19	ST	0.08	1.10
Tampax Blue Box Regular 12S	1.19	T	0.09	1.18
Bodyform Ultra super wing towels 12s	1.19	ST	0.12	1.39

**Table 2:** Top 20 pain products which cover 53% of the total menstrual products sold by the retailer between 30^th^ April 2006 and 16^th^ April 2015.

MP Description	Percentage of Sales	Category	Unit Price	Overall Price
PARA CPLT 16	5.08	Pain Relief Oral	0.04	4.17
VALUE HEALTH PARA CAPLETS 16 16	4.57	Pain Relief Oral	0.02	1.72
PARA CPLT 32	4.32	Pain Relief Oral	0.06	1.32
PA&CO CPL32	4.03	Pain Relief Oral	0.13	1.40
PARA&COD EFF 32	3.63	Pain Relief Oral	0.05	4.39
VALUE HEALTH IBUPROFEN TABS 16	3.60	Pain Relief Oral	0.03	1.60
PARA EXT TAB16	3.43	Pain Relief Oral	0.11	0.63
IBU200MG CPL16	3.30	Pain Relief Oral	0.08	1.09
PARA TABS 16	2.53	Pain Relief Oral	0.03	5.41
PLUS SOL TAB 32	2.41	Pain Relief Oral	0.72	1.65
NUROFEN TABS 16	1.82	Pain Relief Oral	0.07	4.31
NUROFEN PLUS 32	1.70	Pain Relief Oral	0.90	0.38
PARA CAPS 16	1.63	Pain Relief Oral	0.09	1.06
PARA TB 32	1.53	Pain Relief Oral	0.11	1.13
ANADIN EXTRA CPL 16	1.48	Pain Relief Oral	0.13	0.40
SYNDOL CPL30	1.40	Pain Relief Oral	0.36	2.46
PARA & CODEINE TABS 32	1.39	Pain Relief Oral	0.12	1.54
Ibuprofen 200MG Caplets 16	1.35	Pain Relief Oral	0.13	0.66
NUROFEN CAPL 16	1.20	Pain Relief Oral	0.07	0.20
IBUPROFEN 400MG CPLT 48	1.05	Pain Relief Oral	0.35	0.43

For the purposes of this research, a *menstrual pain* transaction is defined as any shopping basket that contains a menstrual item *as well as* at least one pain relief item. The underlying logic herein is that those who suffering from menstrual pain are more likely to purchase menstrual items and pain relief items simultaneously than in isolation. This assumption is of course an instrumental one: related menstrual pain purchases may occur across different baskets, or even across different stores. Equally, despite the likelihood of connection, pain items purchased within a basket may not be directly related to the menstrual products they co-purchased with. As such, the proxy measure we present must not only be normalized against a background of surrounding purchase patterns but must also be understood as a relative measure - and one that is likely a lower bound for true menstrual pain purchasing behaviours overall.

### Estimating the relative prevalence of menstrual pain

The proxy measure we derive for menstrual pain purchases is a probabilistic one, reflecting the propensity of a set of customers to purchase pain items at the same time as menstrual items, compared to their purchase of pain items individually. To calculate this, we define the following probabilities for each store:

- **Propensity of Menstrual Purchase, P**(*M*): The probability that any individual transaction made across a set of customers (and time period) contains a menstrual item. This reflects a proxy measure for the prevalence of menstruation.
- **Propensity of Pain purchases, P**(*P*): The probability that any individual transaction made across a set of customers (and time period) contains a pain item. This reflects a proxy measure for the prevalence of pain.
- **Propensity of Menstrual Pain, P**(*P*|*M*): The conditional probability that a given transaction contains a pain item *given that it already contains a menstrual item*. This provides a naïve proxy measure for the prevalence of menstrual pain.

Note we can also define the propensity of *non*-menstrual pain purchases as **P**(*P*|¬*M*)

As discussed in the previous section, care must be taken not to simply assume that pain and menstrual items that co-occur in a basket are related. In all proxy measures derived from observational data, it is impossible to detect a direct causal effect between menstruation and a pain purchase without direct engagement with an individual themselves. However, confidence of a causal relationship can be strengthened by accounting for surrounding purchase patterns. To this end, we calculate the *ratio* of how likely menstrual pain customers were to purchase a pain product, normalized against their propensity to buy pain products individually:


Menstrual Pain Purchasing Ratio (MPR) = P(P|M) / P(P|¬M) (1)

The MPR proxy measure reflects the relative propensity of a customer (or set of customers) to make a menstrual-pain transaction, normalized against the probability that any of their transactions would contain a pain item anyhow. Note also that this definition does not currently reflect the extent of pain experienced, but merely whether pain alleviation products are more likely to exist within a menstrual basket at all.

To convert transactional data into proxy measures for the relative prevalence of menstruation, pain and menstrual pain across a customer or set of customers (e.g., reflecting a store’s overall custom), we first filter the set of all anonymized loyalty card holders who make at least one transaction involving a menstrual, pain or menstrual *and* pain item over the ten-year period. These subsets are termed *menstrual*, *pain* and *menstrual pain* customers, and their data is then aggregated (allowing for assurance of strict privacy constraints, while maintaining a signal at regional levels). For these customer sets we then calculate the probabilistic measures described in the previous section (*P*(*M*), *P*(*M*), *P*(*M*|*P*) and MPR), and are hence able to label each of the retailers stores across England with a proxy measure relating to menstrual-pain prevalence. In addition to these probability measures, we also determine the top twenty pain and menstrual items most frequently purchased across the retailer to support analysis of the relationship between cost of product and frequency of purchase, and to provide further insight into population level behaviour surrounding menstruation and pain Fig 2.

### Regional analysis and linkages to deprivation

**Regional Features:** To understand variation in relative prevalence of menstrual pain, we first associate all of the proxy measures we derived to each store with the geographical region in which it is situated. While catchment areas for individual stores can vary, the assumption that they serve those regions in which they are embedded generally holds. For this study we employ geographical analysis at the level of *Middle Super Output Support Level* (MSOA) areas. England is delineated into 6506 MSOAs, each with a minimum size of 5,000 residents and 2,000 households and an average population size of 7,800. Regional aggregation of this kind not only adds further protection to individual privacy but also has been shown to predict health outcomes better than individual-level deprivation data. If two stores occur within the same MSOA their results are aggregated. The result is a set of MSOA level *P*(*M*), *P*(*P*), *P*(*P*|*M*) and overall MPR scores.

**Deprivation Features:** To be able to relate menstrual pain ratios to sociodemographics and shed light on the impact deprivation factors have on menstrual pain sales, we leverage the UK Office of National Statistics (ONS) *Indices of Multiple Deprivation* (IMD). The IMD reflects a range of deprivation score, first introduced in a UK government study which measured deprivation at the higher resolution of Lower Layer Super Output (LSOA) levels. IMD scores a range of factors including income, education, crime, living environment, barriers to housing and health. To translate these LSOA level statistics into the larger MSOA geographical levels used in our study, we simply calculate the mean deprivation scores across each LSOA within an MSOA (with MSOAs containing four to five LSOA neighbourhoods on average). This leaves us with a range of mean deprivation factors (e.g., income levels, educational achievements, employment rates) for each MSOA, against which we can correlate menstrual pain proxy measures.

Associations between our probabilistic measures of menstrual-pain purchases, regional deprivation factors (e.g., IMD score, income, education) and population descriptors were assessed using Pearson correlation, consistent with standard practice in epidemiological and population-health research for aggregated area-level data [16]. However, simple linear analyses of this nature have their limitations, especially when sub-populations and non-linearities exist within the data [17]. To address this we also employ a more contemporary, predictive machine learning (ML) approach, described in further detail below. Predictive modelling techniques of this nature are increasingly utilised in population health research to quantify associations, predict outcomes, and interpret feature contributions in complex observational datasetsets [16–21].

### Classification of menstrual and menstrual pain

To further explore the relationship between deprivation and menstrual, pain and menstrual pain transactions this study examines a variety of ML classification models optimised to predict regional menstrual, pain and menstrual pain proxies (our dependent variables) using a set of deprivation and sociodemographic factors as an input feature set (our independent variables).

MSOAs are first classified into “High” or “Low” menstrual, pain and menstrual pain categories (for P(M), P(P), and P(P|M) respectively) based on whether they exceeded the national mean or not. We test a range of classification algorithms, whose performance is validated using 10-fold cross-validation [16]. Investigating the relationship between population health and surrounding sociodemographic, clinical, and behavioural factors in this manner is an increasingly common methodological approach, with applications ranging from forecasting of respiratory disease using retail purchasing data [19] to cardiovascular disease risk prediction [20]. For recent literature reviews in the field, please see Morgenstern et al. [16] and Wiemken & Kelley [18].

Feature importance in resulting models is evaluated using the widely applied SHAP (Shapley Additive Explanations) framework [21], which provides mathematically rigorous and interpretable assessments of the contribution and hence importance of each predictor (independent variable) [20]. This approach is widely used in population health and behavioural analytics to quantify associations and interpret feature contributions in complex observational datasets [19,20].

Our input feature set (independent variables) for each model contains a range of factors drawn the UK’s Indices of National Deprivation alongside four additional demographic variables: *IMD score, Household Income, Electricity Spend, Jobseekers Claimants, Room Occupancy, Population, Population Children, Population Working, Population Older*. Due to the high level of correlation between the ONS’s *income* and the *weekly household income* factors, we exclude the former. Each feature is Z-normalised to lie between zero and one. A range of classification models are examined to explore the relationship space, including Logistic Regression, Random Forest, Support Vector Machines and Neural Network Classifiers. To determine an optimal model, we perform 10-cross fold validation for each of the binarised target variables, reporting resulting accuracy, standard deviation and variable importance analysis.

This study did not involve direct Public and Patient Involvement but benefited from broader engagement within the Digital Footprints Lab. Public consultations on shopping data for health research informed our approach, addressing ethical considerations and data privacy. Insights from these discussions helped shape the study design, and findings will be shared through upcoming public engagement publications.

## Results

### Descriptive analysis

**Fig 1:**
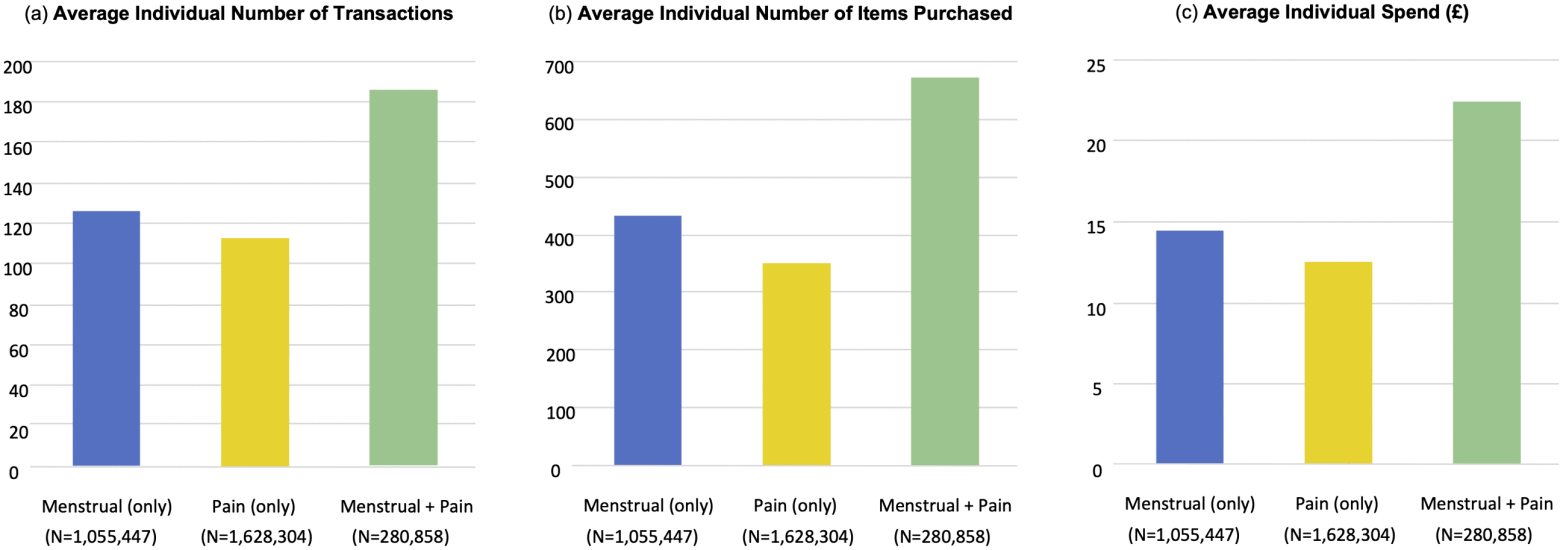
Average (mean) individual summary statistics for Menstrual, Pain and Menstrual Pain customer sets via analysis of transactional logs between 30th April 2006 to 16th April 2015. The number of transactions is representative of the mean total transactions made by each individual customer in each set of customer data of the ten year period. *Number of items* details the mean total number of items purchased per customer in each dataset, again over the ten year period. Individual spend figures reflect the total amount spent by each individual customer featuring in each dataset. Note that *menstrual-pain customers* reflect the intersection of pain customers and menstrual customers.

A descriptive analysis of the transactional dataset, containing the details of all transactions made by loyalty card holders between the 30th April 2006–16th April 2015 is summarised in Fig 1. The mean total number of menstrual transactions per customer is 126 (across 419 menstrual items) reflecting a mean menstrual spend of £1453 over the ten-year period. Overall, 18% of loyalty card customers made a menstrual transaction at the store (1,055,447 individuals). The mean number of pain transactions per customer was 354, with a mean total spend of £1279 over the ten-year period. 31% of loyalty card holders (1,628,304 individuals) made a pain transaction. Importantly, of those customers who made a menstrual transaction, 26.7% were found to have purchased a pain item at the same time (280,858 individuals). Notably menstrual pain customers reflected a more ‘loyal’ shopper to the store and are consequently individuals whose shopping in the dataset is likely more representative of their overall behaviours. In particular, such customers complete 1.47 times more transactions of any kind and generate 1.51 times greater spend than pain customers alone; and similarly complete 1.64 times more transactions and generate 1.72 times more spend than menstrual shoppers alone.

Within the menstrual pain customer set, half of menstrual transactions also included a pain product. For this customer set we calculated the Menstrual Pain Purchasing Ratio (MPR), with a result of 3.9. This translates to the fact that menstrual pain customers are almost four times more likely to purchase a pain product alongside a menstrual product than they were to purchase a pain product on its own.

**Fig 2:**
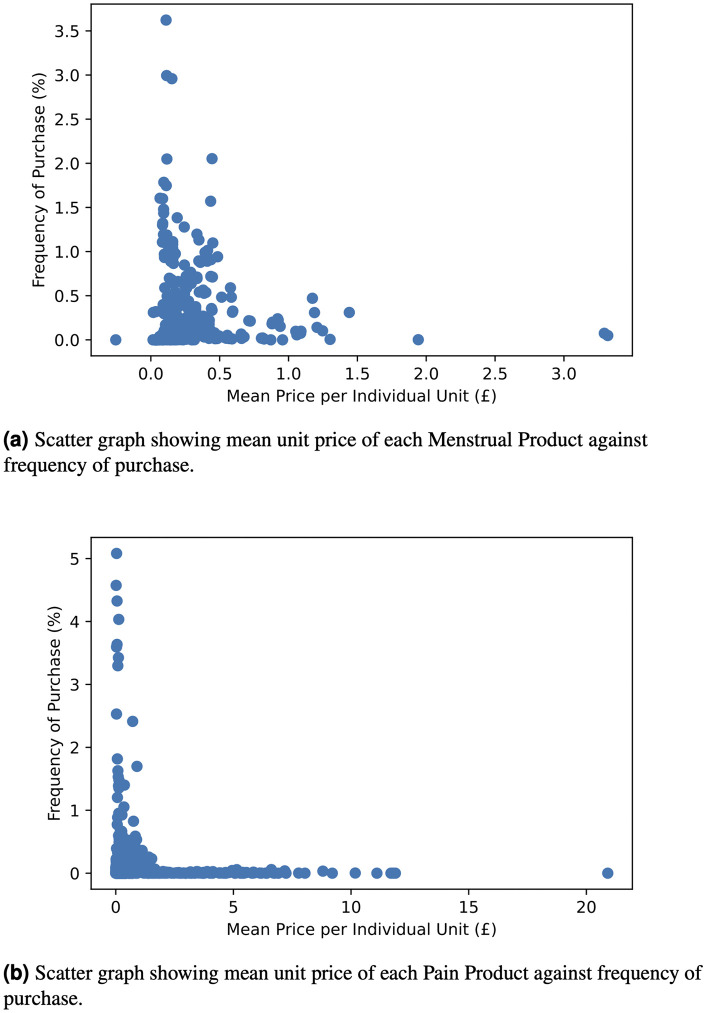
Figure showing the correlation between unit price and frequency of purchase for all menstrual items (a) and pain items (b). There is a stronger correlation between price and frequency of purchase for pain items than for menstrual.

When considering the frequency of purchase of specific products, we found that menstrual pads were purchased more frequently than tampons, with 60% of the top twenty menstrual items being pads and 40% being tampons. A list of the top twenty Menstrual and Pain items are shown in Table 1 and Table 2 respectively. The top twenty menstrual items account for 35% of all menstrual items sold and the top twenty pain items account for 53% of the pain items sold by the retailer which indicates there is greater variation in menstrual items than pain items purchased by loyalty card holders. The relationship between frequency of purchase and cost of product is shown in Fig 2, demonstrating a stronger correlation between prices and purchase frequency for pain items than menstrual indicating that customers show a stronger preference for purchasing cheaper pain items, or indeed not purchasing pain relief at all, than menstrual products.

### Validation of menstrual periodicity within transactional data

To assess the validity of using menstrual transactions as a proxy for the prevalence of menstruation we investigate whether we can detect periodic behaviour around purchase of menstrual products which mirrors the menstrual cycle. Results provided strong empirical evidence of the validity of the signals held within shopping data, with the mode number of days between menstrual transactions across all customers being 28. See below for details.

As one would expect, in our raw dataset there exists high volatility in the mean and median gaps observed between consecutive menstrual purchases – variations that are likely due to a customer irregularly shopping at competing retailers, stocking up in a single store-visit or missing a regular shopping trip due to life circumstances/travel. To account for such variation, we additionally filtered for any consecutive menstrual transactions that lay outside the interval of 21 and 35 days, which is conventionally reported as upper and lower bounds of the normal menstrual cycle (see for example [22]). Of this subset, the mode, mean and median number of days between each menstrual transaction is almost exactly 28 days both for all menstrual customers (N = 691,133, Mode Internal = 28, Mean Interval ± std = 368 ± 485, Median Interval = 171, Max Interval = 3268, Min Interval = 0) and filtered menstrual transactions (N = 218,516), Mode Internal = 28, Mean Interval ± std = 28.07 ± 3.83, Median Interval = 28, Max Interval = 35, Min Interval = 21). Summary statistics for average interval (in days) between transactions for individual menstrual customers. *All menstrual customers* indicate the subset of loyalty card holders who had made at least two menstrual transactions over the ten-year period. *Filtered menstrual transactions* indicates the subset of transactions made by all menstrual customers where the interval between transactions was between 21 and 35 days.

Our results strongly evidences that the average interval between menstrual transactions is 28 days, which corroborates the periodic nature of the menstrual cycle, and provides strong empirical evidence for the potential use of transactional data as a proxy for menstruation behavioural analyses.

### Geographical disparity of menstrual pain purchases

To assess the extent to which menstrual pain sales vary geographically we use a simple probabilistic model to aggregate individual store sales into the relevant Middle Layer Super Output Area (MSOA). From this aggregation, each probability measure detailed in the methods section is calculated for each MSOA. The Pearson correlation coefficient between each regional probability measure with each deprivation proxy measure, aggregated at the MSOA level, are shown in Table 3. All p-values associated with coefficients shown in Table 3 are below 0.05.

**Table 3:** Table showing Pearson correlation of Index of Multiple Deprivation (IMD) score, Office National Statistics deprivation factors and population descriptors with Total Transactions per MSOA; P(M) for each MSOA; P(P) for each MSOA and P(P|M) for each MSOA.

	Total Transactions	Menstruation Proxy P(M)	Pain Proxy P(P)	Menstrual Pain Proxy P(P|M)
IMD	0.09	-0.07	-0.26	-0.31
Household Income	0.04	0.40	0.16	0.36
Electricity Spend	-0.26	0.01	0.34	0.26
Jobseekers Claimants	0.17	0.08	-0.25	-0.17
Room Occupancy	0.16	0.09	-0.04	0.00
Population All	0.17	0.21	-0.02	0.05
Population Children	0.00	0.12	-0.16	-0.02
Population Working	0.34	0.33	-0.17	0.02
Population Older	-0.30	-0.28	0.38	0.08

Each probability measure is negatively correlated with overall IMD score, yet IMD is positively correlated with the number of all transactions per MSOA region. As a higher IMD score indicates a higher level of deprivation in a given MSOA, the more deprived an area, the greater the number of overall transactions yet the fewer the number of menstrual and menstrual pain sales. As it would be expected, MSOAs with a higher proportion of elderly population were negatively correlated with menstrual and menstrual pain sales but positively correlated with pain sales. Table 3 also shows the correlation between each probability measure and mean income, electricity spend, population proportion claiming jobseekers’ allowance and mean household room occupancy.

**Fig 3:**
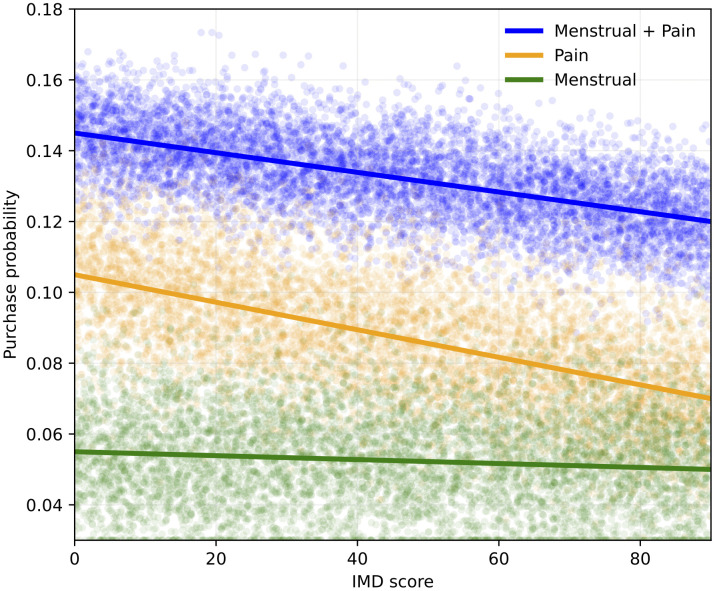
Figure shows the relationship between IMD score (x-axis) and menstrual (green), pain (orange) and menstrual pain (blue) probability (y-axis). Each MSOA is represented by an individual point and solid lines show the line of best fit for each measure. The Figure shows that there is a negative correlation between IMD and each of the probability measures such that MSOAs with high level of deprivation have lower menstrual, pain and menstrual pain probabilities. This relationship is strongest for probability of pain transactions.

Fig 3 shows the relationship between IMD score and probability of menstrual, pain and menstrual pain for every individual MSOA as well as the line of best fit for each of the probability measures. There is a negative correlation between IMD score and all three of the probability measures related to menstruation indicating that the most deprived regions in England are associated with the lowest number of menstrual pain sales.

### Regional menstrual pain classification

To further evaluate the relationship between deprivation and menstrual pain sales we evaluate the effectiveness of the proxy deprivation and demographic factors in individual MSOA as predictors for each probability measure. We design a binary classification task by defining each MSOA as a “High” or “Low” transaction area where “High” MSOAs have menstrual, pain, or menstrual pain probability greater than the national mean. As reported by Morgenstern et al, 2020 [16], the most common machine learning classification algorithms used to predict population health outcomes are Neural Networks, Support Vector Machines, Random Forests and Logistic Regression. In order to find a best-of-class prediction model (whose workings we can then interrogate), we therefore optimize each of these classification model classes using each deprivation and demographic factors as training features and the associated “High” or Low” binary transaction label as the target to be predicted. Following a 10-fold cross-validation exploration of the parameter space (using a grid search), optimal model results for each class are shown in Table 4.

**Table 4:** Classification accuracy scores with standard deviation for the binary transformed P(M), P(P) and P(P|M) under a variety of classification models using the feature set of Table 3. The most successful model on all three target variables was the neural network classifier.

Model	P(M)	P(P)	P(P|M)
Logistic Regression	0.70 ± 0.16	0.69 ± 0.08	0.63 ± 0.11
Random Forest	0.70 ± 0.16	0.67 ± 0.06	0.62 ± 0.06
Support Vector	0.70 ± 0.16	0.69 ± 0.09	0.63 ± 0.12
Neural Network	0.83 ± 0.06	0.80 ± 0.07	0.80 ± 0.06

To assess the predictive performance of each of the candidate classifiers we selected classification accuracy as our assessment metric: the sum of the correctly classified MSOAs divided by the total number of MSOAs. The neural network classifier proved the most successful in correctly predicting each MSOA for each probability measure with a predictive accuracy of 0.83, 0.8 and 0.8 for P(M), P(P) and P(P|M) respectively. This result indicates there is a strong predictive relationship between our features of deprivation (IMD score, IMD employment, IMD education, IMD Crime, IMD Barriers, IMD Environment, ONS income, ONS, Electricity, ONS jobseekers, ONS Room Occupancy) and the classification of each MSOA in terms of its menstrual pain sales.

To understand which of these features was most influential in this classification model we perform feature importance via the SHAP framework, first introduced by Lundberg et al. [23]. SHAP is built using the game theoretic Shapley Value to provide mathematically rigorous evaluation of which input features (independent variables) are most important to a given classification.

**Fig 4:**
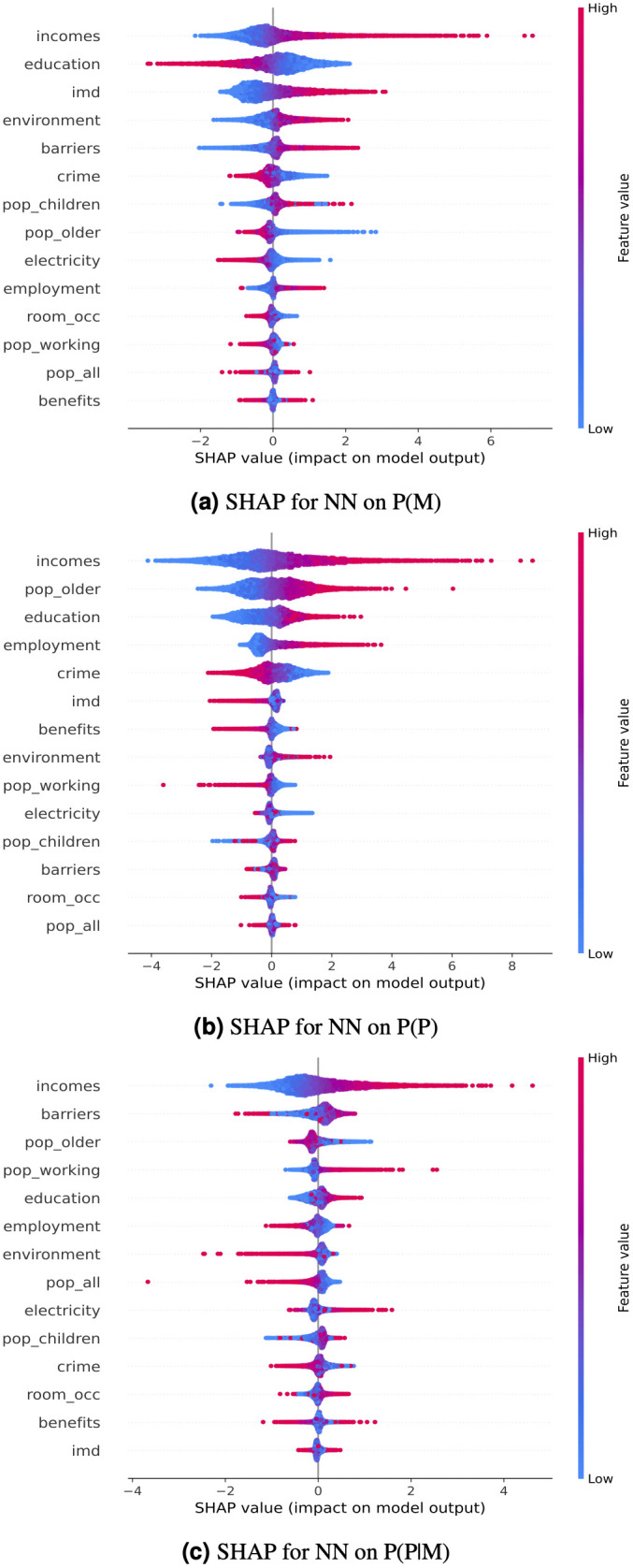
SHAP feature importances that have been evaluated on the neural network classifier for each of the binariseda) P(M) b) P(P) and c) P(P|M). The features are sorted from biggest impact on the model (top) to least influential (bottom). The horizontal axis indicates the extent to which a given feature supported the classification of high transaction MSOAs (bar extending towards the right) and a low transaction MSOAs (bar extending towards the left). weekly income was the most influential feature for all three classification tasks and that higher regional weekly income are associated with higher levels of P(M) P(P) and P(P|M). correlation between regional income and the number of menstrual, pain and menstrual pain transactions. Most importantly, loyalty card customers in the MSOAs with the lowest national average incomes were 32% less likely to make a menstrual pain purchase than those in the MSOAs with the highest average incomes.

Fig 4 shows the SHAP value importance for each feature in the classification of the binary transformed probability measures. Red and blue shading indicates a high or low feature value respectively. The horizontal axis indicates the extent to which a given feature supported the classification of high transaction MSOAs (bar extending towards the right) and a low transaction MSOAs (bar extending towards the left).

For example, in the P(M) classification task, a high income value was strongly influential in the correct classification of MSOAs with high menstrual item purchasing propensities, whereas high education deprivation (high education factor IMD score) was strongly influential in classifying MSOAs with low purchasing propensities for menstrual products. For each target variable, income was the most influential feature in the resulting classification task, demonstrating a strong positive correlation between regional income and the number of menstrual, pain and menstrual pain transactions. Most importantly, loyalty card customers in the MSOAs with the lowest national average incomes were 32% less likely to make a menstrual pain purchase than those in the MSOAs with the highest average incomes.

## Discussion

Our study is the first to using a novel data source to overcome this data scarcity by using menstrual and pain purchases as a proxy for prevalence of menstruation, pain and menstrual pain respectively. Our results motivate the use of transactional data as a proxy for underlying menstrual symptoms, showing that the mode interval between menstrual transactions for each menstrual customer is 28 days which coincides with the length of the average menstrual cycle. This result motivates the future use of transactional data for further analysis of menstrual health management.

Results show that a large proportion of those buying menstrual products are purchasing pain relief simultaneously evidencing significant prevalence of menstrual pain in England with 26.7% of menstruating loyalty card holders purchasing a menstrual and pain item simultaneously. This is an important finding which is very likely an underestimate of prevalence of menstrual pain as it would not capture those who do not (or cannot afford to) self-medicate, as well as those who manage menstrual pain in a different way (e.g., hormonal contraceptives, prescribed pain medication, using local application of heat).

Additionally, there is significant regional variation in menstrual pain sales where the MSOAs associated with the highest national levels of deprivation (highest IMD scores) are also associated with the lowest probability of menstrual pain transactions. Previous research in dysmenorrhea has primarily focused on how biological differences affect the level of pain felt by certain groups. In [24] it is argued that dysmenorrhea is largely unaffected by external societal structures. Our results show that there are clear differences between menstrual pain sales across deprived and non-deprived areas. Our SHAP feature analysis shows that there is a strong predictive relationship between average regional income and menstrual pain sales. Stores located in regions associated with the lowest household income are a third less likely to make a menstrual pain sale than stores located in regions with the highest household income. The more deprived a region is, the fewer menstrual pain transactions are made.

Prior research investigating the association of socioeconomic status with dysmenorrhea have been limited to small qualitative studies focused on one particular demographic, e.g., a subset of Bangladeshi or Turkish women [25]. It has been repeatedly shown that there is no association between menstrual problems and socioeconomic status [25–27]. In contrast, when studying the impact socioeconomic status has on the ability to manage dysmenorrhea, it has been empirically shown that inability to purchase menstrual pain relief reflects and re-enforces socioeconomic disparity where the inability to afford menstrual products and thus manage bleeding and pain symptoms causes anxiety from the resulting increased risk of stigmatisation [28,29].

However, it is clear from our results that there is a link between regional financial deprivation and the number of menstrual pain transactions being made. This result should motivate further study to establish whether this variation in menstrual pain transactions is due to the inability of those in more deprived areas to afford period pain medication, serving as empirical evidence health inequalities in England.

The use of transactional data in this study is subject to limitations: Our dataset only includes transactions of loyalty card users of one pharmaceutical retailer, there may be a higher number of menstrual pain sufferers who purchase menstrual and pain items at alternative retailers where they could be cheaper. This calls for additional further analysis of menstrual pain transactions to understand whether those suffering from dysmenorrhea are not able to medicate it due to financial barriers or whether they medicate it using other shops to the one we analysed the data from. Additionally, our proxy measure for menstrual pain is subject to an assumption that sufferers of menstrual pain purchase pain and menstrual items at the same time, it included customers who may be purchasing both items as part of a routine shop (i.e., a weekly shop) yet it does not include customers who may purchase their pain and menstrual items at separate times. Further, some customers might be purchasing menstrual and menstrual pain items for other people, e.g., mother purchasing for their daughter.

**Key Messages**

**What is already known on this topic**

Menstrual pain is a common but often overlooked by health systems concern affecting many individuals globally. Existing research highlights its negative impact on daily activities, including school and work attendance. However, the intersection between menstrual pain and economic hardship remains underexplored, particularly in how financial constraints influence the management of menstrual pain.

**What this study adds**

This study provides new evidence that economic hardship significantly affects the management and experience of menstrual pain. Findings indicate high prevalence of menstrual pain in England. Statistically significant differences exist between deprived and less deprived neighbourhoods of England in terms of proportion of menstrual pain sales. The findings suggest that menstrual pain is not always just a medical issue but also a socioeconomic concern.

**How this study might affect research, practice or policy**

The study highlights the need for greater awareness and policy interventions to address the high prevalence of menstrual pain as well as socioeconomic dimensions of menstrual pain. Public health initiatives should incorporate menstrual pain relief as part of broader efforts to improve health equity. Additionally, workplace and educational policies should recognize and accommodate menstrual pain-related challenges, particularly for those in economically disadvantaged groups.
